# Idiopathic Sudden Sensorineural Hearing Loss: Is Hyperbaric Oxygen Treatment the Sooner and Longer, the Better?

**DOI:** 10.3390/jpm12101652

**Published:** 2022-10-05

**Authors:** Chun-Shih Chin, Tsai-Yun Lee, Yi-Wen Chen, Ming-Feng Wu

**Affiliations:** 1 Division of Pulmonary and Critical Care Medicine, and Hyperbaric Oxygen Therapy Center, Department of Internal Medicine, Taichung Veterans General Hospital, Taichung 40705, Taiwan; 2 Division of Chest Medicine, Department of Internal Medicine, Taichung Veterans General Hospital, Taichung 40705, Taiwan; 3 Department of Medical Laboratory Science and Biotechnology, Central Taiwan University of Science and Technology, Taichung 40601, Taiwan

**Keywords:** idiopathic sudden sensorineural hearing loss, hyperbaric oxygen treatment, prognosis, pure-tone audiometry

## Abstract

(1) Background: We aimed to evaluate hearing benefits from hyperbaric oxygen (HBO) therapy in patients with Idiopathic Sudden Sensorineural Hearing Loss (ISSHL). (2) Methods: We performed a retrospective analysis of chart reviews on patients with ISSHL between Jan 2016 and Dec 2021. All patients were referred to receive HBO therapy by the department of Ear, Nose and Throat (ENT). Hearing gain was assessed based on pure-tone audiometry (PTA). Data were analyzed for 102 patients after 1 to 5 therapy sessions, and for 46 patients after 6 to 10 therapy sessions. (3) Results: After 1–5 HBO sessions, patients (N = 102) showed an improvement in 45 (44.1%) of the patients (*p* < 0.000). Also, improvements were found with patients showing different grades of ISSHL: 11 (26.8%) with slight-moderate, 11 (40.7%) with severe, and 23 (67.6%) with profound ISSHL. Significant treatment effects were found at different affected frequencies, especially the low frequency range. After 6–10 HBO sessions, patients (N = 46) showed similar treatment effects as after 1–5 HBO sessions, but no additional improvement. Moreover, patients who received HBO treatment within 12 days showed improvement effects 6.484 times greater (*p* < 0.000) compared with those who received treatment after 13 days. (4) Conclusions: The improvement of HBO therapy on ISSHL was significant after 1–5 sessions, with larger improvements for those suffering more serious symptoms. Further adding more HBO treatment sessions to 6–10, no further improvement was found. Patients starting HBO therapy within 12 days of ISSHL showed 6.484 times greater improvements compared with those starting HBO therapy later.

## 1. Introduction

Idiopathic sudden sensorineural hearing loss (ISSHL) is considered by otologists as a true otologic emergency whose pathogenesis is uncertain [1]. It is associated with either total or partial loss of a patient’s hearing function. This disease is common and is defined when sensorineural hearing loss at a minimum of 30 decibels occurring in at least three consecutive frequencies and lasting at least 3 days [2,3]. The patient typically first notices symptoms upon awakening and describes an aural fullness/blockage. They may also experience tinnitus (usual), dizziness, or vertigo. According to the World Health Organization (WHO), it is the most common cause of disability globally, and the third leading cause of years of productivity lost due to disability [4], and the 15th leading cause of burden of disease, and is projected to move up to 7th by the year 2030, especially in high-income countries [1,5].

Earlier, salvage therapy with intra-tympanic steroid (ITS) and/or hyperbaric oxygen (HBO) therapy was added if patients did not adequately benefit from systemic steroid therapy. One study showed that ITS and HBO therapy produced similar improvements in ISSHL patients [6]. The other’s trial used HBO therapy as salvage therapy for ISSHL patients and showed some benefits in hearing improvement. Better results could be expected in patients with severe hearing loss and low frequencies [2,7,8]. The cochlea of inner ear is an organ that depends on adequate levels of oxygen in the blood. However, due to the protected location of the cochlea in the temporal bone, the blood supply to this organ is very limited. Blood is supplied to the cochlea mainly through the labyrinthine arteries. Cochlear hair cells have high oxygen consumption and poor tolerance to hypoxia, which is why the inner ear is prone to circulatory changes. ISSHL appears to be characterized by hypoxia in the perilymph, in the scala tympani and the organ of Corti. Therefore, a mechanism that may play a role in ISSHL appears to be related to hypoxia. Therefore, the use of HBO therapy in patients with ISSHL is a reasonable choice [5]. HBO therapy may improve the oxygen supply to the inner ear, thereby improving the patient’s hearing, but the effect is better if early HBO therapy is combined with other treatment modalities [3,9]. From Systematic review and Meta-analysis trials, the hearing gain at five contiguous frequencies was significantly higher in the HBO therapy and systemic steroids [2,10]. HBO therapy did not improve results when it was started late as salvage therapy [11,12,13]. 

According to European Committee of Hyperbaric Medicine (ECHM), it would also be reasonable to use HBO therapy as an adjunct to oral steroid (OS) and/or ITS in patients with severe or profound hearing loss (≥70 dB), but only in cases presented not later than one month after symptoms onset. Some studies suggest that salvage therapy with HBO is ineffective. However, adjuvant therapy is effective [5,14]. In clinical practice, the current standard treatment for ISSHL is a tapered course of high-dose systemic steroid therapy [1,2]. More recently, ITS treatment has emerged and become more popular with otolaryngologists. This is an interesting option due to the absence of unfavorable side effects [endocrine problems (i.e., diabetes dysregulation), osteoporosis or weight gain, which are known in the systemic steroid treatment [2]. Treatment protocols aim to decrease the inflammatory state of the inner ear while increasing the blood supply and oxygenation. However, ethically we cannot simply compare hyperbaric oxygen therapy with the current standard treatment for ISSHL. Now, we have treated ISSHL patients with a tapered course of oral high-dose corticosteroids and/or ITS treatment at our department of Ear, Nose and Throat (ENT), while adding adjunctive HBO therapy referred from our ENT department as soon as possible.

The prognosis of the disease is believed to be better that the sooner the ISSHL treatment including HBO therapy is initiated and a prolonged duration therapy of at least 1200 min (about 20 sessions) is added [10]. HBO therapy did not improve results when it was started late [11] and was not efficient when performed three weeks after the onset of the ISSHL [12]. The most important factors affecting prognosis were the duration from disease onset to treatment time and hearing loss severity [15], but a retrospective study showed HBO therapy ineffective in severe and profound patients [16].

In October 2011, the Undersea Hyperbaric Medicine Society (UHMS) added ISSHL to its list of approved indications [8] and in April 2016, the tenth European consensus conference on hyperbaric oxygen treatment was supported by sufficiently strong evidence [14]. However, in Taiwan, the Central Health insurance Bureau does not pay for this indication. Patients still need to receive HBO therapy, but do so at their own expense. Many patients care treatment effects more than their own expense. We hope that our presentation of objective data provides a clearer reference regarding the recommendation of HBO therapy in patients with ISSHL, through the results of this study analysis.

This study aimed to evaluate the hearing gain efficacy from HBO therapy in ISSHL patients. By analyzing the extent of improvement after HBO therapy (hearing gain) in the duration of ISSHL onset time to HBO therapy initial time, HBO therapy sessions, gender, age, affected ear side, and the initial hearing loss severity, we aim to find its relevance. Can slight to moderate (≤60 dB HL), severe (61 to 80 dB HL) and profound (≥81 dB HL) ISSHL patients be improved with HBO therapy? Will the duration from ISSHL onset time to HBO therapy affect the prognosis? Is the benefit of HBO therapy greater with more HBO therapy sessions?

## 2. Materials and Methods

### 2.1. Study Design, Setting and Population

We performed a retrospective analysis of chart review on patients with ISSHL between Jan 2016 and Dec 2021. All patients were referred to us from the department of ENT, and the otolaryngologist was based on the patient’s condition, medical history, and related examinations, including hearing test (first pure-tone audiometry (PTA) data) to confirm ISSHL diagnosis before pharmacological and HBO therapy. If the patient was diagnosed with ISSHL, the standard treatment (oral steroid and/or ITS) was started in the ENT department, and referral to us for adjuvant HBO therapy. A tapered course of oral high-dose corticosteroids with prednisolone (1 mg/kg/day) given as 30 mg twice daily for two days, then reduced by 20 mg per two days, the final 7th day 10 mg once daily, a total treatment period of seven days, and/or ITS was given at our ENT department. Hearing was assessed through PTA (Instrument: GSI 61 Clinical Audiometer. Instrument S/N: 20010300, GS07095). Three PTA assessments were conducted: before, after 1–5, and after 6–10 HBO therapy sessions. Data on ISSHL onset time, age, gender, affected ear side, HBO therapy initial time, and treatment sessions were recorded.

Those patients excluded were due to incomplete PTA data (no data before and after HBO therapy). Those patients who had initial hearing loss PTA < 25 dB, which was incompatible with WHO classification of the severity of hearing impairment were also excluded. Four additional patients were also excluded due to their young ages (<20 years old). Patients with ear injuries to both sides were excluded as they likely had more comorbidities, longer history of the disease, and did not respond well to therapy. The study was approved by the hospital’s Institutional Review Board and Ethics Committee (approval number: CE22261A).

### 2.2. HBO Therapy Sessions

**Figure 1:**
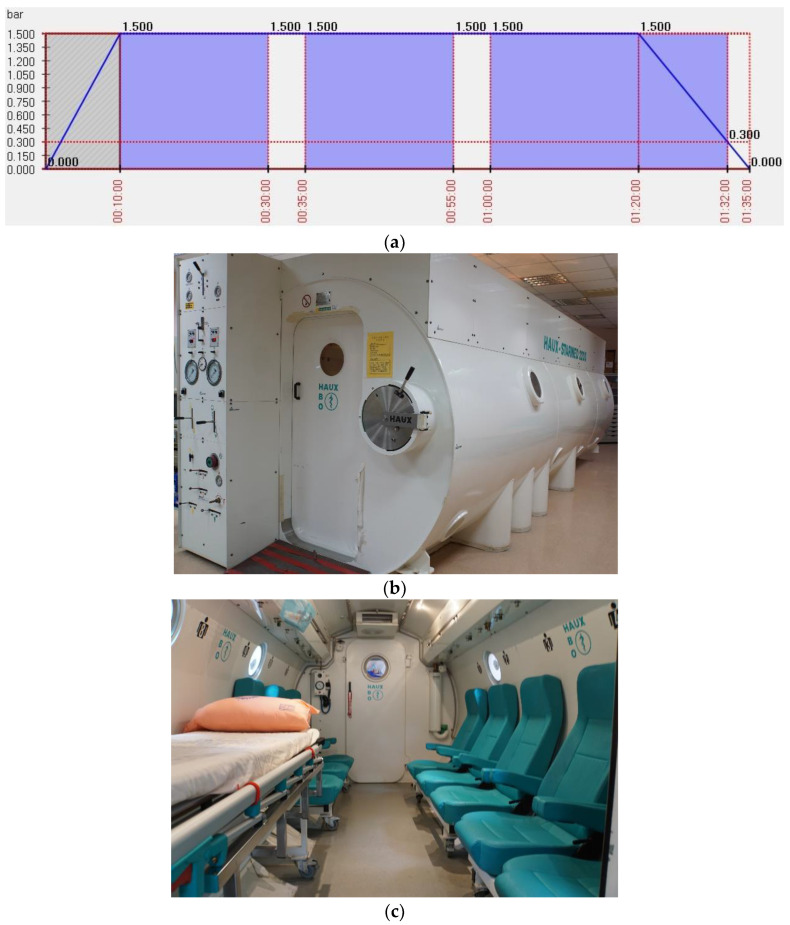
Hyperbaric oxygen treatment protocol and the equipment. (**a**) A standard 2.5 atmospheres absolute, 95-min treatment protocol (two, five-minute air breaks per session. Total 60 min treatment time in 2.5ATA per session); (**b**) Hyperbaric oxygen treatment chamber appearance in Taichung Veterans General Hospital, Taiwan; (**c**) Hyperbaric oxygen treatment chamber internal appearance.

All patients’ HBO therapy followed a standard 2.5 atmosphere absolute (ATA) 95-min regimen, with two 5-min air breaks per session (Figure 1a–c), in accordance with our ENT department standard ISSHL treatment protocol. Patients signed self-pay agreements with the HBO therapy consent forms before treatment.

To avoid middle ear barotrauma, patients were instructed before HBO therapy to equalize middle ear pressure by themselves, and to evaluate the efficacy of both the Valsalva and Toynbee maneuvers. In cases where patients were unable to equalize their middle ear pressures, they were referred to our ENT department to receive a procedure of tympanic membrane puncture. With appropriate education and evaluation, only 30% of patients experienced pain at the eardrum during the HBO pressurizing step [8]. When such pain occurred, pressurizing speed was reduced, and time allowed for patients to adjust to pressure changes. A few patients underwent myringotomy. From this single center 20-year analysis, a significantly low seizure rate was achieved with the implementation of a 5-min air break. Assessing and defining the appropriate patient/HBO therapy profile is therefore useful in minimizing risks of central nervous system oxygen toxicity [17]. No patient in this study had oxygen toxicity seizure side effects.

### 2.3. The Measurement of Hearing

The outcome of the hearing was measured in terms of the levels of hearing gain in PTA. By the WHO classification of the severity of hearing impairment, hearing loss was defined as grade 0: no impairment (25 dB or better), grade 1: slight impairment (26–40 dB HL), grade 2: moderate impairment (41–60 dB HL), grade 3: severe impairment (61–80 dB HL), and grade 4: profound impairment including deafness (81 dB or higher HL) [4]. Because of the small sample size in this study and the definition of ISSHL, we separated patients into three groups according to their initial severity of hearing loss: slight to moderate (≤60 dB HL), severe (61 to 80 dB HL) and profound (≥81 dB HL).

Because of its relative simplicity, and its ability to be performed without any knowledge of prior hearing function or a normally hearing contralateral ear, improvement in PTA change was defined as one of the three: hearing “improvement” with a gain of ≥10 dB, “steady” with a gain of between 0 and 10 dB, and “deterioration” with a gain of ≤0 dB. While hearing recovery rate (HRR) was defined as the ratio of the difference after HBO therapy for hearing gain divided by initial hearing level minus contralateral hearing level.

### 2.4. Statistical Analyses

For continuous variables, data were expressed as median with Q1 and Q3 intervals: Q1, the median of the lower half of the data, and Q3 the median of the upper half of the data. Categorical variables were represented by numbers (percentages). For the comparisons of continuous variables, pair differences were conducted with Wilcoxon signed-rank tests, while groups difference was performed with Mann–Whitney U test. Categorical variables were compared with the Chi-Square test.

To assess the effect of intervention time (IT) on the improvement of HBO therapy, the optimal cut-off with Youden index was performed using the Receiver-operating characteristic (ROC) curve [18]. Different grades of hearing impairment and potential confounding factors were merged into the cut-off IT of HBO therapy to assess the hearing improvement using multiple logistic regression. Statistical analyses were performed using the SPSS software version 18.0 (SPSS Inc., Chicago, IL, USA) with statistical significance set at *p* < 0.05.

## 3. Results

A total of 148 ISSHL patients (81 male, 67 female) were studied. Their median age was 52.5 years old. Dizziness/vertigo was reported in 53 patients (35.8%), and tinnitus in 87 patients (58.8%). Among them, 102 patients were for only 1–5 sessions, and 46 were for additional 6–10 sessions. Table 1 shows more of their characteristics, including intervention time (IT) of disease onset to HBO therapy initial time, baseline PTA data, affected hearing frequencies and steroid therapy status.

For HBO therapy of only 1–5 sessions on 102 patients, their initial PTA evaluations showed improvements in 45 patients (44.1%) (*p* < 0.000). Among them 11 (26.8%) had slight-moderate impairment, 11 (40.7%) had severe impairment, and 23 (67.6%) had profound impairment. The median hearing recovery rate was 21.7%. (Table 2). Those with more serious symptoms showed greater improvement effects. Significant hearing gains (*p* < 0.05) were found over all tested frequencies (250 Hz, 500 Hz, 1k Hz, 2k Hz, 4k Hz, 8k Hz). For patients with hearing loss involving multiple frequencies, significant HBO effects were found especially in low frequencies.

**Figure 2:**
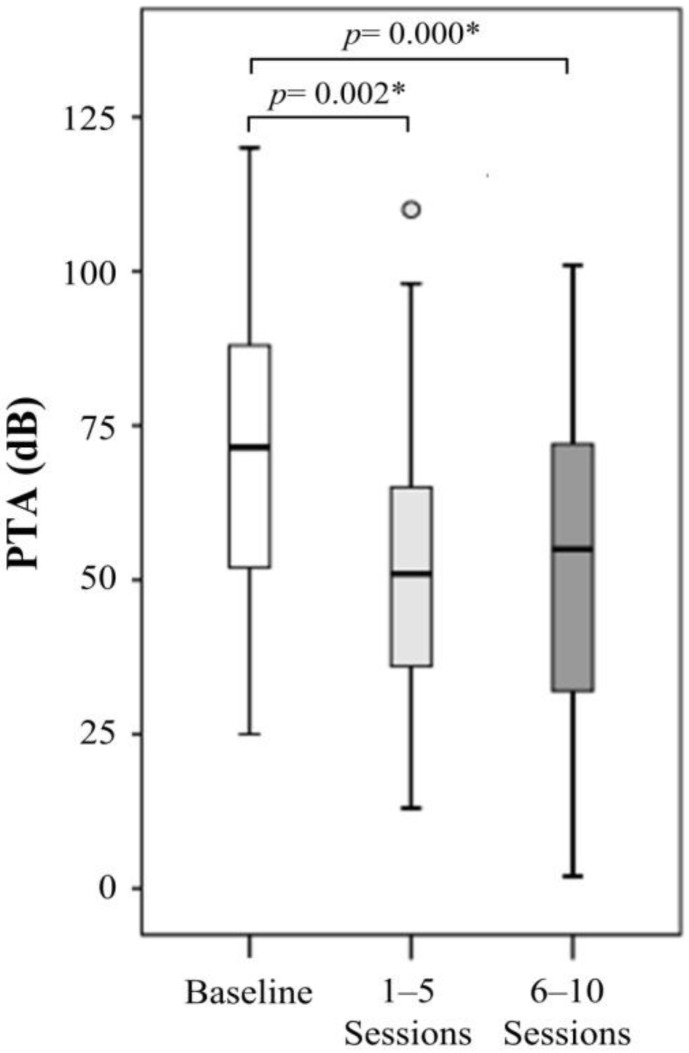
The treatment effect for HBO therapy 6–10 sessions. The measurement of 1–5 sessions and 6–10 sessions were completed after HBO therapy 1–5 sessions and 6–10 sessions, respectively, for patients with HBO therapy 6–10 sessions. *p*-value was determined by Wilcoxon signed-rank test. *: *p* < 0.05.

For HBO therapy of additional 6–10 sessions on 46 patients, no further improvement in PTA data was found over those of 1–5 sessions over all affected frequencies (Table 2). In brief, no additional benefits were found with longer HBO treatments (Figure 2). We noted that the numbers of PTA measurements were not identical between those treated for 1–5 sessions and 6–10 sessions (n = 20 and 46, respectively). The discrepancy in measurement numbers likely led to some statistical bias.

**Figure 3:**
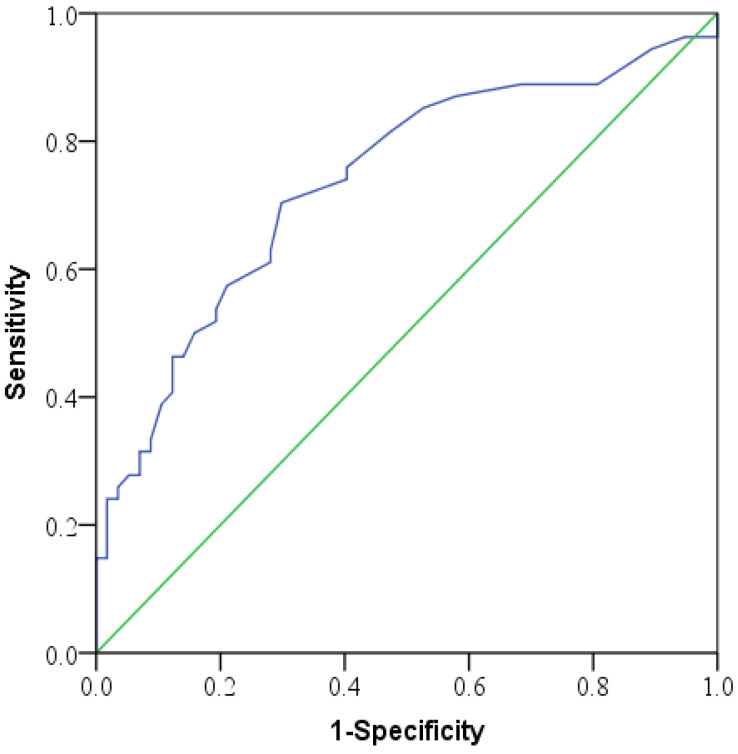
The ROC curve of intervention time for treatment improvement on pure-tone audiometry (PTA) for patients with HBO therapy 1–5 sessions. The area under the curve was 0.736.

Based on the ROC analysis, the effective IT was 12.5 days (Figure 3). The sensitivity was 0.704 and the specificity was 0.702 at the cut-off level. The age, gender, affected ear, dizziness/vertigo, tinnitus, steroid used status and the IT of the ISSHL onset to HBO therapy were tested for confounding factors on hearing improvement across different affected frequencies. A low correlation existed with age at frequencies of 2, 4, and 8 kHz. A moderate correlation was found with the intervention time at all affected frequencies (Table 3). However, when excluding the effect of age, we found a 2.267-fold improvement in those with severe hearing impairment when compared with those with slight-moderate hearing impairment. Such a difference was not statistically significant (*p* = 0.132). Similarly, when profound hearing impairment was compared with slight-moderate hearing impairment, we found a 5.034-fold improvement, which was statistically significant (*p* = 0.004). Results compared between those receiving HBO within 12 days and those after 13 days (or the intervention time from ISSHL onset to HBO therapy start time), the success rate was 6.484 times greater (*p* < 0.000) (Table 4).

We analyzed the intervention time between the onset of ISSHL and the start of HBO therapy in each group in patients who received only their first HBO therapy 1–5 sessions (N = 102) and those who received an additional second HBO therapy 6–10 sessions (N = 20).

Patients who received HBO therapy within 12 days had a hearing gain of 20.5 dB and a hearing recovery rate of 41.6%, compared with patients who received HBO therapy after 13 days with a hearing gain of only 3 dB and a hearing recovery rate of 9.4%, but in the comparison of the HBO therapy 1–5 sessions and an additional second HBO therapy 6–10 sessions, neither hearing gain nor hearing recovery rate have seen any further benefits, either HBO therapy within 12 days or after 13 days (Table 5).

In addition, we analyzed the intervention time between the onset of ISSHL and the start of HBO therapy (N = 46) in each group containing patients who received HBO therapy 6–10 sessions directly. Patients who received HBO therapy within 12 days had a hearing gain of 32 dB and a hearing recovery rate of 52.2%, while those who received HBO therapy after 13 days had a hearing gain of only 5 dB and a hearing recovery rate of 9.7%. In comparisons of the HBO therapy 1–5 sessions (Table 5), hearing gain and hearing recovery rates were better whether HBO therapy was performed within 12 days or after 13 days (Table 6), but not statistically difference.

## 4. Discussion

According to the 10th European Consensus Conference on Hyperbaric Medicine, HBOT is strongly indicated as a primary treatment for ISSHL. That is supported by strong evidence with some in favor of randomized controlled trial (RCT) and ample expert consensus (Level of evidence B) [14]. Various forms of treatment have become widely accepted as being beneficial in most cases, and not providing such treatments to patients could conceivably be viewed as unethical. For ISSHL patients, the three most promising treatments are corticosteroids (ITS), vasodilators, and hyperbaric oxygen therapy. Of these, only hyperbaric oxygen has sufficient RCTs to have a positive metanalysis Cochrane review [4]. In this study, all patients received a tapered course of oral high-dose corticosteroids (74.3%) and/or ITS (76.4%) therapy, and a small number of patients only received ITS therapy because they were afraid of the side effects of systemic steroids. In addition, a small number of patients were afraid of injections, so they only received oral steroid therapy. The patients were then referred for HBO therapy, so we could analyze whether it would be better to start HBO therapy earlier and more HBO therapy sessions.

In our study, no patient was left untreated. Patients came from and were treated at our ENT department. We specifically tested their pure-tone audiometry levels three times: before, after 1–5, and after 6–10 HBO therapy sessions. We also recorded their intervention times (from disease onset to the start of HBO therapy). Several studies reported HBO therapy has no improvement on hearing when started late [11,12,13]. The effectiveness of HBO therapy is time-dependent and decreases with an increasing delay in administration. Generally, it is recommended to start with therapy as early as possible [5]. Other studies found that early HBO therapy combined with other clinically approved modalities (systemic steroid, ITS) provides better results than the same modalities administered on their own [1,3,9]. It is known from previous clinical studies that different mechanisms of treatment can be used together to achieve better results. Steroids are used to fight inflammation and HBO therapy is used to promote oxygenation. In our study, all patients received a tapered course of oral high-dose corticosteroids and/or ITS. We found results differed with intervention times within 12 days or after 13 days. Early intervention before 12 days had an odds ratio of the better treatment effect of 6.484. In patients who received only their first HBO therapy 1–5 sessions (N = 102) and those who received an additional second HBO therapy 6–10 sessions (N = 20), HBO therapy when applied early within 12 days of disease onset (a hearing gain of 20.5 dB and a hearing recovery rate of 41.6%), the improvement effect was significantly better than after 13 days of disease onset (a hearing gain of 3 dB and a hearing recovery rate of 9.4%). In patients (N = 46) who included HBO therapy 6–10 sessions directly, the hearing gain was 32 dB and the hearing recovery rate was 52.2% within 12 days of HBO therapy, while the hearing gain of patients receiving HBO therapy after 13 days was 5 dB and the hearing recovery rate was 9.7%. Therefore, patients should be encouraged to start HBO therapy as soon as possible.

One study compared HBO therapy and ITS injection on hearing gain on ISSHL patients, after attempts with primary treatment have failed [6]. The study found similar improvements between ITS and HBO therapy. However, for now, we have been using these treatment modalities altogether. Other treatment modalities when combined at the same time, effects are likely better.

Some studies reported that HBO therapy is better for those with initial severe hearing loss [2,7,8,10]. Some studies indicate that patients with moderate ISSHL are improved by HBO therapy along with oral steroids, but HBO therapy is ineffective for patients with severe and profound hearing losses [16,19]. Another study reported that HBO therapy plus systemic steroid and ITS treatment show no gain in hearing regardless of the severity in hearing loss [20]. In summary, HBO effects might be better for patients with severe and profound hearing losses or with slight to moderate hearing losses. In our study, we found that HBO therapy had better effects for those with the worse initial hearing loss.

One systematic review and meta-analysis study concluded that the longer the HBO treatment duration (>1200 min, ~20 sessions), the more effective the response will be [10]. In our study, we divided HBO treatment duration into 1–5 and 6–10 sessions. The number of HBO therapy sessions was the patient’s decision. Patients elected either more HBO sessions to improve efficacy or fewer sessions to save money. HBO therapy hearing gain with the number of HBO therapy sessions showed no further improvement beyond 5 sessions in our study. However, the intervention time in including patients who received HBO therapy 6–10 sessions directly, in comparisons with HBO therapy 1–5 sessions, whether HBO therapy was given within 12 days or after 13 days, hearing gain (32 dB vs. 22 dB within 12 days, 5 dB vs. 3 dB after 13 days) and hearing recovery rates (52.2% vs. 41.9% within 12 days, 9.7% vs. 9.4% after 13 days) were both better, but not statistically different. Possible explanations include that patients who are satisfied with their hearing gain after the first HBO therapy 1–5 sessions may discontinue an additional second HBO therapy 6–10 sessions because the national health insurance system did not cover the HBO therapy. If the patients received additional sessions, they paid more for the HBO therapy. If so, the complex case undergoes further treatment and the outcome of the additional HBO therapy may be poorer; or, associated with that it is a relation to the small number of patients treated with 6–10 sessions (N = 46, 31.1%). Only 20 patients had the completed three PTA tests: before, after 1–5, and after 6–10 HBO therapy sessions. More patients are needed to confirm better effects from longer HBO sessions.

A limitation of this study is that the original design was to track the data of PTA once after the patient has completed 5 HBO therapy sessions and then the data of PTA again after 10 HBO therapy sessions, and then see if there is further hearing gain. However, in the real world, the number of HBO therapy is the patient’s decision. Moreover, the PTA test requires the cooperation of the patient. Therefore, it is not possible to collect a full dataset for every patient. In our study, measurements of 1–5 sessions and 6–10 sessions were completed after HBO therapy 1–5 sessions and 6–10 sessions were 20 and 46, respectively. Given the small sample size, it is difficult to conclude firmly whether it is more effective doing more HBO therapy sessions. Also, bilateral hearing loss usually occurs gradually over time. But in rare situations, it can occur suddenly. One systematic review reported that those involving bilateral hearing loss is less than 5% [2]. The causes of bilateral hearing loss are related to congenital condition, medication, loud sound exposure, physical damage, ear infection, and aging. Because bilateral hearing loss does not typically occur suddenly (rare cases) and is often caused by known reasons, it is not included in this study.

## 5. Conclusions

We found that in the adjunctive HBO therapy for the ISSHL patients, after 1–5 sessions, 45 (44.1%) of them showed improvement (*p* < 0.000). Among them, 11 (26.8%) had slight-moderate impairment, 11 (40.7%) had severe impairment, and 23 (67.6%) had profound impairment before HBO treatment. The median hearing recovery rate was 21.7%. Comparing those with profound hearing impairment and those with slight-moderate hearing impairment, we found a 5.034-fold greater improvement, which was statistically significant (*p* = 0.002). Those with more serious ISSHL showed a greater HBO effect. 

Besides, hearing gain showed a significant difference (*p* < 0.05) in all different affected frequencies (250 Hz, 500 Hz, 1 kHz, 2 kHz, 4 kHz, 8 kHz). There were significant HBO effects in before-after data, especially in low frequencies. 

The effective time point of the intervention time was 12.5 days. Patients who started HBO therapy within 12 days had 6.484 times the greater effect, which was statistically significant (*p* < 0.000). HBO therapy within 12 days had better effect in hearing gain and hearing recovery rate, compared with HBO therapy after 13 days. After HBO therapy of 6–10 sessions showed no further improvement in hearing when compared with those after therapy of 1–5 sessions. Moreover, the intervention time in patients who received HBO therapy 6–10 sessions, in comparisons with HBO therapy 1–5 sessions, whether HBO therapy was given within 12 days or after 13 days, hearing gain and hearing recovery rates were both better.

## Figures and Tables

**Table 1:** The basic characteristics of enrolled patients.

	All (n = 148)	Only 1–5 Sessions (n = 102)	Additional 6–10 Sessions (n = 46)
Age (years old)	52.5 (39.3, 60.0)	52.5 (37.0, 61.0)	52.5 (45.8, 60.0)
Sex			
Male	81 (54.7)	56 (54.9)	25 (54.3)
Female	67 (45.3)	46 (45.1)	21 (45.7)
Dizziness/Vertigo	53 (35.8)	39 (38.2)	14 (30.4)
Tinnitus	87 (58.8)	59 (57.8)	28 (60.9)
Intervention time (day)	13.0 (7.0, 28.8)	13.0 (7.0, 29.0)	13.0 (5.0, 24.0)
Baseline			
PTA (dB)	68.0 (47.0, 93.0)	68.0 (45.0, 93.5)	71.5 (50.8, 88.0)
slight-moderate	59 (39.9)	41 (40.2)	18 (39.1)
severe	37 (25.0)	27 (26.5)	10 (21.7)
profound	52 (35.1)	34 (33.3)	18 (39.1)
250 Hz (dB)	60.0 (45.0, 85.0)	60.0 (45.0, 86.3)	55.0 (40.0, 81.3)
500 Hz (dB)	65.0 (50.0, 90.0)	67.5 (50.0, 90.0)	65.0 (50.0, 95.0)
1K Hz (dB)	70.0 (50.0. 95.0)	70.0 (45.0, 95.0)	72.5 (53.8, 90.0)
2K Hz (dB)	70.0 (46.3, 90.0)	70.0 (45.0, 91.3)	70.0 (50.0, 90.0)
4K Hz (dB)	75.0 (56.3, 95.0)	75.0 (50.0, 96.3)	75.0 (60.0, 96.3)
8K Hz (dB)	82.5 (60.0, 100.0)	80.0 (60.0, 100.0)	85.0 (60.0, 100.0)
Treatment time	5.0 (5.0, 9.8)	5.0 (5.0, 5.0)	10.0 (10.0, 10.0)
Right/left side injured	72 (48.6)/76 (51.4)	51 (50.0)/51 (50.0)	21 (45.7)/25 (54.3)
ITS/OS used	113 (76.4)/110 (74.3)	80 (78.4)/77 (75.7)	33 (71.7)/33 (71.7)
Any steroid used	148 (100.0)	102 (100.0)	46 (100.0)

PTA: pure-tone audiometry; HBO: hyperbaric oxygen; ITS: Intra-tympanic steroid; OS: oral steroid.

**Table 2:** The measurement for HBO therapy only 1–5 sessions (n = 102). The measurement for HBO therapy with additional 6–10 sessions (n = 46).

	Baseline	1–5 Sessions	Difference	HRR (%)	Improvement (%)	*p*-Value
PTA (dB)	68.0 (45.0, 93.5)	57.0 (34.5, 72.0)	7.0 (0.0, 23.0)	21.7 (0.0, 45.7)	45 (44.1)	0.000 *^α^
slight-moderate (n, %)	41 (40.2)				11 (26.8)	0.002 *^β^
severe (n, %)	27 (26.5)				11 (40.7)	
profound (n, %)	34 (33.3)				23 (67.6)	
250 Hz (dB)	60.0 (45.0, 86.3)	50.0 (25.0, 70.0)	5.0 (0.0, 26.3)		49 (48.0)	0.000 *^α^
500 Hz (dB)	67.5 (50.0, 90.0)	55.0 (25.0, 70.0)	10.0 (0.0, 35.0)		55 (53.9)	0.000 *^α^
1K Hz (dB)	70.0 (45.0, 95.0)	60.0 (35.0, 76.3)	5.0 (0.0, 25.0)		48 (47.1)	0.000 *^α^
2K Hz (dB)	70.0 (45.0, 91.3)	60.0 (33.8, 75.0)	5.0 (0.0, 20.0)		38 (37.3)	0.000 *^α^
4K Hz (dB)	75.0 (50.0, 96.3)	70.0 (43.8, 80.0)	0.0 (0.0, 15.0)		37 (36.3)	0.000 *^α^
8K Hz (dB)	80.0 (60.0, 100.0)	75.0 (45.0, 90.0)	0.0 (0.0,11.3)		33 (32.4)	0.000 *^α^
	**Baseline (n = 46)**		**1–5 Sessions (n = 20) ^ε^**		**6–10 Sessions (n = 46)**	
PTA (dB)	71.5 (50.8, 88.0)		51.0 (33.0, 66.0)		55.0 (31.0, 72.8)	
250 Hz (dB)	55.0 (40.0, 81.3)		40.0 (25.0, 65.0)		40.0 (20.0, 60.0)	
500 Hz (dB)	65.0 (50.0, 95.0)		45.0 (35.0, 75.0)		55.0 (23.8, 70.0)	
1K Hz (dB)	72.5 (53.8, 90.0)		60.0 (35.0, 75.0)		60.0 (30.0, 75.0)	
2K Hz (dB)	70.0 (50.0, 90.0)		60.0 (20.0, 75.0)		60.0 (35.0, 70.0)	
4K Hz (dB)	75.0 (60.0, 96.3)		65.0 (45.0, 85.0)		65.0 (50.0, 80.0)	
8K Hz (dB)	85.0 (60.0, 100.0)		75.0 (55.0, 90.0)		75.0 (53.8, 91.3)	

PTA: pure-tone audiometry; HRR: hearing recovery rate; ^α^: Wilcoxon signed-rank test between baseline and cycle 1–5; ^β^: Chi-square test between baseline and cycle 1–5; ^ε^: The measurement was completed after 1–5 sessions for patients with HBO therapy 6–10 sessions; *: *p* < 0.05.

**Table 3:** An analysis of potential factors for the HBO treatment effect on hearing.

	PTA (dB)	250 Hz (dB)	500 Hz (dB)	1K Hz (dB)	2K Hz (dB)	4K Hz (dB)	8K Hz (dB)
Age ^κ^	−0.297 *	−0.281	−0.296	−0.235	−0.343 *	−0.343 *	−0.290 *
Intervention time ^κ^	−0.499 *	−0.387 *	−0.487 *	−0.465 *	−0.425 *	−0.390 *	−0.238 *
Sex (male: female) ^λ^	0.348	0.181	0.462	0.916	0.072	0.269	0.172
Ear (right: left) ^λ^	0.265	0.307	0.241	0.392	0.154	0.153	0.203
Dizzness/Vertigo (yes: no) ^λ^	0.759	0.207	0.983	0.147	0.688	0.810	0.992
Tinnitus (yes: no) ^λ^	0.729	0.562	0.584	0.945	0.770	0.818	0.377
ITS used (yes: no) ^λ^	0.525	0.841	0.819	0.708	0.193	0.905	0.848
Oral steroid (yes: no) ^λ^	0.291	0.530	0.630	0.351	0.021 *	0.045 *	0.130

PTA: pure-tone audiometry; ITS: Intra-tympanic steroid; ^κ^: Spearman correlation; ^λ^: the *p*-value for difference with Mann-Whitney test. *: *p*< 0.05.

**Table 4:** Multiple logic regression for the improvement of HBO treatment for PTA.

Factors	Odds Ratio (95% CI)	*p*-Value
Age (per 1% increase)	0.976 (0.948–1.005)	0.270
Grades of hearing impairment		
Severe vs. slight-moderate	2.267 (0.781–6.585)	0.132
Profound vs. slight-moderate	5.034 (1.844–13.743)	0.002 *
Intervention time		
≦12 days vs. ≧13 days	6.484 (2.748–15.296)	0.000 *

* p< 0.05.

**Table 5:** The comparison of treatment effect for patients with the first HBO therapy 1–5 sessions.

	From Any Sessions (N = 122)	From Therapy 1–5 Sessions (N = 102)	From Therapy 6–10 Sessions (N = 20)	*p*-Value
Intervention time ≦ 12 days	N = 56	N = 44	N = 12	
Difference of PTA (dB)	20.5 (7.3, 38.5)	22.0 (5.5, 39.0)	18.5 (8.5, 38.5)	0.734 *^λ^*
Hearing recovery rate (%)	41.6 (16.9, 78.8)	41.9 (23.1)	30.1 (8.9, 71.2)	0.293 *^λ^*
Improvement	40 (71.4)	31 (70.5)	9 (75.0)	0.533 ^β^
Intervention time ≧13 days	N = 66	N = 58	N = 8	
Difference of PTA(dB)	3.0 (−2.0, 10.0)	3.0 (−1.3, 8.5)	3.0 (−3.0, 16.8)	0.890 *^λ^*
Hearing recovery rate (%)	9.4 (−2.7, 27.1)	9.4 (−1.6, 28.9)	12.9 (−17.0, 22.9)	0.639 *^λ^*
Improvement	17 (25.8)	14 (24.1)	3 (37.5)	0.336 ^β^

PTA: pure-tone audiometry; ^λ^: the *p*-value was determined with Mann-Whitney test; ^β^: the *p*-value was determined with Chi-square test.

**Table 6:** The effect of intervention time for patients with HBO therapy 6–10 sessions.

	All (N = 46)	Intervention Time≦ 12 Days (N = 21)	Intervention Time ≧13 Days (N = 25)	*p*-Value
Difference of PTA (dB)	13.5 (2.8, 33.0)	32.0 (17.0, 50.5)	5.0 (−0.5, 9.5)	0.000 *^λ^
Hearing recovery rate (%)	27.4 (4.4, 55.3)	52.2 (28.4)	9.7 (0.0, 26.6)	0.000 *^λ^
Improvement	25.0 (54.3)	19.0 (90.5)	6.0 (24.0)	0.000 *^β^

PTA: pure-tone audiometry. ^λ^: the *p*-value was determined with Mann-Whitney test; ^β^: the *p*-value was determined with Chi-square test; *: *p* < 0.05.

## Data Availability

Not applicable.
